# Nanoscale Control of Amyloid Self-Assembly Using Protein Phase Transfer by Host-Guest Chemistry

**DOI:** 10.1038/s41598-017-06181-4

**Published:** 2017-07-18

**Authors:** Tae Su Choi, Hong Hee Lee, Young Ho Ko, Kwang Seob Jeong, Kimoon Kim, Hugh I. Kim

**Affiliations:** 1 Department of Chemistry, Korea University, Seoul, 02841 Republic of Korea; 2 Department of Chemistry, Pohang University of Science and Technology (POSTECH), Pohang, 37673 Republic of Korea; 3 Division of Metrology for Quality of Life, Korea Research Institute of Standards and Science, Daejeon, 34113 Republic of Korea; 4 Center for Self-assembly and Complexity, Institute for Basic Science, Pohang, 37673 Republic of Korea

## Abstract

Amyloid fibrils have recently been highlighted for their diverse applications as functional nanomaterials in modern chemistry. However, tight control to obtain a targeted fibril length with low heterogeneity has not been achieved because of the complicated nature of amyloid fibrillation. Herein, we demonstrate that fibril assemblies can be homogeneously manipulated with desired lengths from ~40 nm to ~10 μm by a phase transfer of amyloid proteins based on host-guest chemistry. We suggest that host-guest interactions with cucurbit[6]uril induce a phase transfer of amyloid proteins (human insulin, human islet amyloid polypeptide, hen egg lysozyme, and amyloid-β 1–40 & 1–42) from the soluble state to insoluble state when the amount of cucurbit[6]uril exceeds its solubility limit in solution. The phase transfer of the proteins kinetically delays the nucleation of amyloid proteins, while the nuclei formed in the early stage are homogeneously assembled to fibrils. Consequently, supramolecular assemblies of amyloid proteins with heterogeneous kinetics can be controlled by protein phase transfer based on host-guest interactions.

## Introduction

Diverse amyloid proteins self-assemble to fibrous quaternary structures called amyloid fibrils^1, 2^. The structures of amyloid fibrils are commonly *β*-sheet-rich, long, and unbranched, regardless of the amyloid protein^3^. This supramolecular architecture of amyloid fibrils has recently emerged as a scaffold with diverse functionalities^4–10^. Hence, modulation of the fibril morphology has also been attempted to obtain controlled amyloid scaffolds^11–13^. However, the complexities in amyloid fibrillation, including heterogeneous assembly kinetics, hinder tight control of the fibril length^14^. Thus, the next worthwhile challenge is to modulate the fibril length with a low heterogeneity based on a rational strategy.

Amyloid fibrillation occurs by a nucleation-growth mechanism (Supplementary Fig. S1)^15–17^. The fibrillation is initiated with the nucleation of monomeric proteins; then, the formed nuclei interact with monomers and elongate into amyloid fibrils. The elongation process (*k*_el_ = ~10^4^–10^7^ M^−1^ s^−1^) is faster than the nucleation process (*k*_nu_ = ~10^–2^–10° M^−1^ s^−1^)^18^ because the nuclei, once formed, provide a templated structure to the monomers in the elongation process^19^. Both nucleation and elongation can occur simultaneously during the whole fibrillation process^20^. Hence, the nuclei formed in the early stage are elongated to longer fibrils, while other nuclei are formed in the late stage^20, 21^. Consequently, the distribution of the fibril lengths becomes highly polydispersed^14^. Thus, the suppression of extra nucleation during elongation of the nuclei formed in the early stages can be a strategy toward controlling the fibril length and heterogeneity.

**Figure 1:**
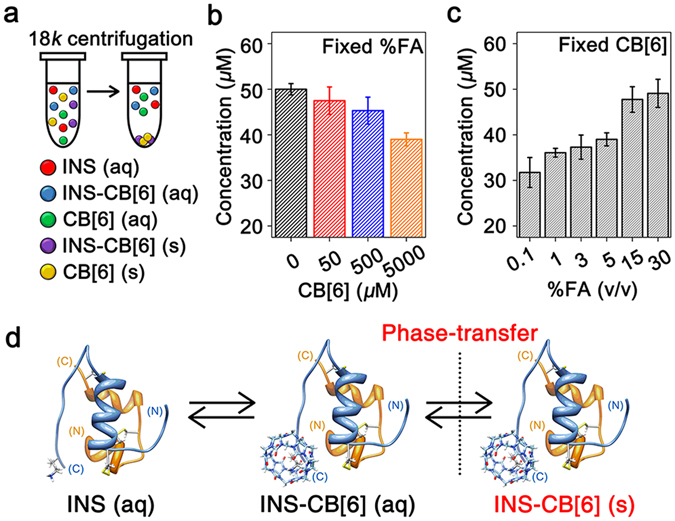
Phase transfer of INS by CB[6]. (**a**) Representation for centrifugation of an INS-CB[6] solution. (**b**) Concentration changes of INS in the supernatant with various amounts of CB[6] in a 5% FA solution. (**c**) Concentration changes of soluble INS in the supernatant with variable %FA with 5 mM CB[6]. (**d**) Phase transfer of INS between the soluble state and the insoluble state.

Herein, we suggest a supramolecular strategy for the precise control of amyloid fibrils of various proteins with desired lengths and high homogeneity. We employed protein phase transfer mediated by host-guest chemistry to control the self-assembly of amyloid proteins. Host-guest chemistry between proteins and synthetic receptors has been widely applied in protein recognition^22^, function regulation^23^, protein assembly^24^, and amyloid inhibition^25, 26^. We previously reported the inhibition strategy of amyloid fibrillation using host-guest chemistry between cucurbit[7]uril (CB[7]) and phenylalanine residues (Phe)^25^. CB[7] bound on Phe sterically hinders protein-protein interactions of amyloid proteins, thereby inhibiting fibrillation processes^25^. In the present study, we utilized cucurbit[6]uril (CB[6], Supplementary Fig. S2) to modulate the fibrillation processes of representative amyloid proteins (human insulin, human islet amyloid polypeptide, hen egg lysozyme, and amyloid-*β* 1–40 & 1–42)^2^. CB[6] is a macrocyclic host molecule constructed from six glycoluril repeating units; it binds to a lysine residue (Lys) in proteins and is barely soluble in water (*e.g*. 20 μM in pure water)^27–31^. Notably, amyloid proteins in the soluble state form a normal host-guest complex with CB[6] at Lys, but they are phase-transferred to the insoluble state when CB[6] exceeds its solubility limit (Fig. 1). We applied this phenomenon in the suppression of the extra nucleation during amyloid fibrillation. Consequently, the fibril length could be controlled for fibrils from tens of nanometres to nearly ten micrometres in length with low polydispersity indices (PDIs ≈ 1.3–1.6).

## Results and Discussion

### CB[6]-induced phase transfer of proteins

We initially confirmed that human insulin (INS, Supplementary Fig. S2)—a well-defined model system for amyloid fibrillation^25, 32^— exists as a monomer in formic acid (FA) using solution small-angle X-ray scattering (SAXS). The radius of gyration (*R*_g_) of INS (0.76 mg/mL in 1% FA) is 10.8 Å from the Guinier analysis of the scattering profile of INS (Supplementary Fig. S3), which corresponds to the *R*_g_ of the INS monomer^33^. The zero-angle scattering intensity (*I*(0)) of the scattering profile was further compared with those of standard proteins (Supplementary Fig. S3), supporting that the average molecular weight of INS agrees with the monomeric state (M_w_ ~ 5.8 kDa). Although INS can exist in various multimeric states^32, 34^, low pH conditions (*e.g*. in acetic acid) stabilize INS in the monomeric state^33^. Thus, we also expect that the low pH (pH ~2.3 for 1% FA) induces the monomeric state of INS. Then, the host-guest interactions between CB[6] and the INS monomer in FA were examined using native polyacrylamide gel electrophoresis and electrospray ionization mass spectrometry (ESI-MS) (Supplementary Fig. S4). The binding between CB[6] and Lys of INS was confirmed by isothermal titration calorimetry and tandem mass spectrometry (MS^2^) (Supplementary Figs S4 and S5). These results indicate that CB[6] forms a complex with INS through a host-guest interaction at Lys, as previously reported^29, 35^.

We further examined the effect of CB[6] on INS in solutions when the amount of CB[6] exceeded its solubility limit. The solubility of CB[6] is very low in water (~20 μM), and increases with the ratio of FA in solution (Supplementary Table S1)^31^. We monitored the concentrations of the dissolved INS monomer in solutions with various amounts of CB[6] and %FA (*v*/*v*). The solutions were centrifuged at 18,000 × g to remove any insoluble aggregates (Fig. 1a), and the concentration of the INS monomer in the supernatant was measured by UV absorption at 280 nm. The concentration of INS (50 μM) decreases as the amount of CB[6] increases in 5% FA (Fig. 1b), and the concentration of INS (50 μM) increases as the %FA increases (*i.e*. the solubility of CB[6] increases) with 5 mM CB[6] (Fig. 1c). These results indicate that CB[6] causes a phase transfer of INS from a soluble to insoluble state.

We expect that the host-guest interaction of the INS-CB[6] complex in the soluble state is followed by CB[6]-mediated phase transfer of INS (Fig. 1d). Briefly, CB[6] in the soluble state forms a host-guest complex with INS in solution, while CB[6] in the soluble state is generated by the equilibrium shift from CB[6] in the insoluble state. Then, the phase transfer of the INS-CB[6] complex occurs when the concentration of the INS-CB[6] complex is saturated in solution. As the amount of CB[6] over its solubility limit increases in the fixed %FA solution, the phase transfer of INS-CB[6] seems to be further promoted by coprecipitation with excess CB[6] in the insoluble state (Fig. 1b). When the %FA increases in solution, the total amount of CB[6] in the insoluble state decreases by the interaction with FA. Thus, the coprecipitation of INS-CB[6] complexes with insoluble CB[6] might be suppressed as the %FA increases (Fig. 1c). Furthermore, the solvation of CB[6] by FA may also compete against the interaction with INS in solution. Consequently, the formation of the INS-CB[6] complex in solution is disrupted by FA; thereby, the amount of phase-transferred INS also decreases as the %FA increases (Fig. 1c). These characteristics of CB[6] were applied to control the nucleation kinetics, for which the concentration of the monomer is significant and whereby the amyloid fibrils can be manipulated with desired lengths and homogeneities.

### Modulation of fibril lengths with low polydispersity by CB[6]

**Figure 2:**
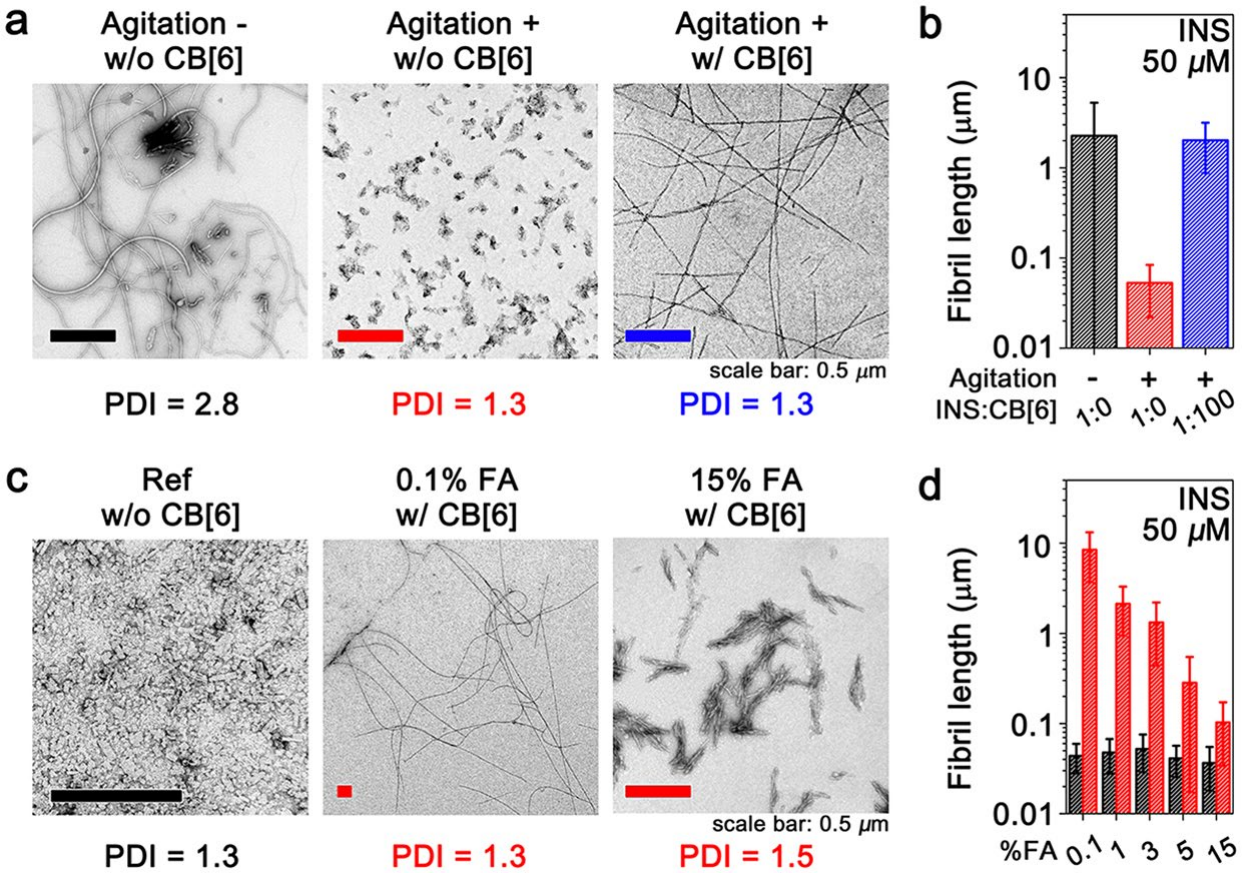
Modulation of INS fibrils by CB[6]. (**a**) TEM images of INS fibrils formed in 5% FA (INS = 50 μM and CB[6] = 5 mM). (**b**) Average lengths of INS fibrils (lengths and PDIs are available in Supplementary Table S2; fibril length distributions are presented in Supplementary Fig. S8). (**c**) TEM images of INS fibrils in variable %FA (INS = 50 μM, CB[6] = 500 μM). Ref is the representative image of INS fibrils formed without CB[6]. All fibrils were prepared with agitation at 200 rpm. (**d**) Average fibril lengths determined from the TEM images in (**c**) (fibril lengths and PDIs are available in Supplementary Table S3).

We examined the effect of CB[6] on the formation of INS fibrils. INS fibrils were prepared from a 50 μM INS solution in 5% FA at 50 °C. We calculated the average length of 1,500 fibrils using transmission electron microscopy (TEM) and quantified the distribution of the fibril lengths based on the PDI values (Supplementary Text). Without any agitation, the fibril lengths of INS (2.3 ± 3.0 μm) were broadly dispersed with a PDI value of 2.8 (Fig. 2a), indicating heterogeneous formation of INS fibrils. Next, we agitated the INS solution at 200 rpm, and obtained short fibrils (53 ± 31 nm) with a PDI of 1.3 (Fig. 2a). This implies that agitation enhances the homogeneity of the fibrils, but fibril elongation is minimal. In the presence of CB[6], we observed no significant fibril formation without agitation. However, in the presence of CB[6] with agitation, the fibril length increases as the concentration of CB[6] increases (Fig. 2b and Supplementary Fig. S6). For example, a hundred-fold increase of CB[6] with respect to INS ([CB[6]] = 5 mM) increases the fibril length by two orders of magnitude (2.0 ± 1.1 μm) while maintaining a low PDI (1.3). The thickness of the fibrils is independent of the agitation and CB[6] concentration (Supplementary Fig. S7). These results indicate that amyloid fibrils with desired lengths and low PDIs can be prepared using agitation and CB[6]. Since significant changes in the fibril lengths are observed when the amount of CB[6] exceeds its solubility limit in 5% FA (80 μM in 5% FA)^31^ with agitation, we expect that this phenomenon is correlated from the effect of the phase transfer by CB[6] observed in Fig. 1.

We further tested the effect of CB[6] with respect to its solubility limit (Supplementary Table S1); thus, we investigated the effect of varying the %FA on the controllable length of the amyloid fibrils while maintaining a low PDI (Fig. 2c and Supplementary Fig. S9). The morphologies of the INS fibrils under each %FA condition were examined without and with CB[6]. The amount of CB[6] (500 μM) in a solution exceeds its solubility limit at each %FA studied, except for 15% FA (CB[6] solubilities: 20 μM in 0.1% FA, 80 μM in 5% FA, and 550 μM in 15% FA)^31^. The fibril lengths without CB[6] (37–53 nm) are similar to that in the 5% FA solution. The fibril lengths with CB[6] increase from tens of nanometres (37 ± 19 nm) to almost ten micrometres (9.8 ± 5.1 μm) as the %FA decreases (Fig. 2d), while the PDI values remain within 1.3–1.6. Overall, our observations indicate that the lengths of the fibrils with a low PDI can be manipulated by controlling the amount of CB[6] above its solubility limit in solution with continuous agitation.

### CB[6]-mediated kinetic control in the fibril assembly

**Figure 3:**
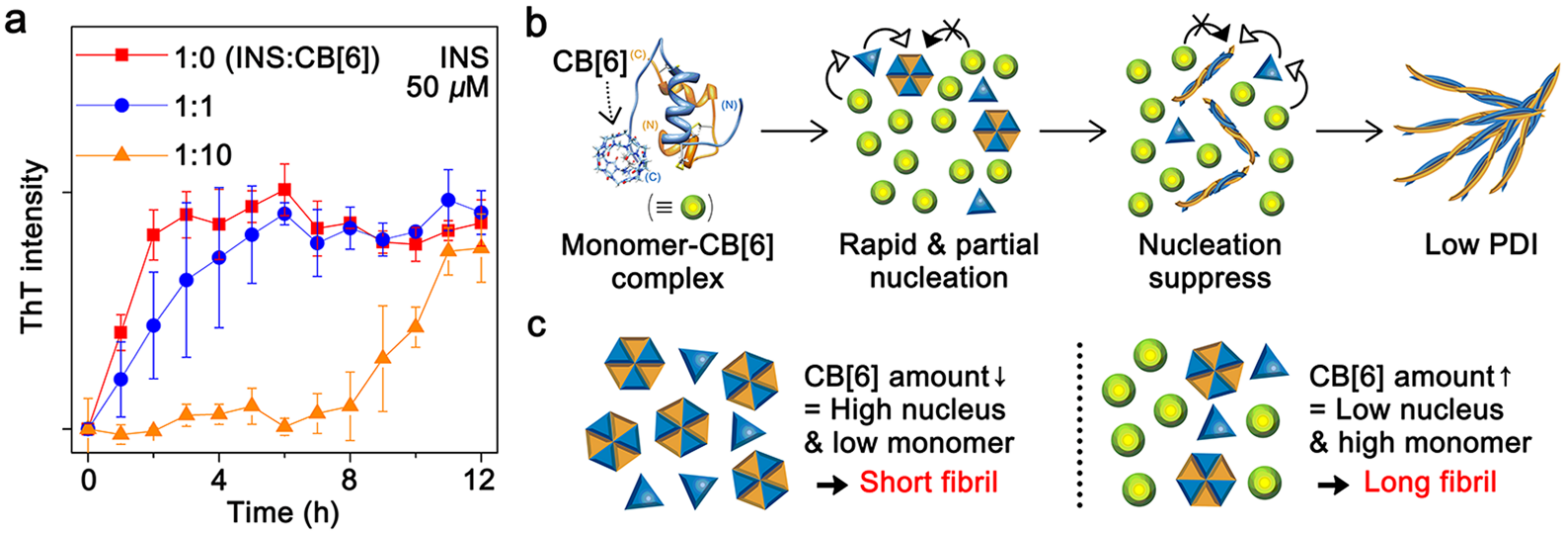
Fibrillation mechanism controlled by CB[6]. (**a**) Thioflavin T (ThT) assay for the kinetics of INS fibrillation with various amounts of CB[6] in solution (INS = 50 μM). The 5 mM CB[6] condition was excluded because of the turbidity of CB[6] in the solution. (**b**) Schematic representation for CB[6]-mediated kinetic control in nucleation. (**c**) Correlation between fibril length and monomer:nucleus ratio. As the amount of CB[6] decreases (left) or increases (right), the fibril length is shortened or lengthened, respectively.

From the observations in Figs 1 and 2, we expect that the agitation and transfer of INS by CB[6] during fibrillation are crucial to control the fibril length and low polydispersity. To support this hypothesis, we monitored the effects of agitation (Supplementary Fig. S10) and CB[6] (Fig. 3a) on the fibrillation kinetics at 5% FA using a thioflavin T (ThT) assay. The assay in Supplementary Fig. S10 suggests that agitation of the INS solution reduces the fibrillation lag phase compared to the control group without agitation. The fibrils formed with agitation are shorter and less polydispersed (53 ± 31 nm, PDI = 1.3) than those formed without agitation (2.3 ± 3.0 μm, PDI = 2.8), which implies that collision events induced by agitation enhance the overall nucleation rate^18, 36, 37^, leading to homogeneous and rapid formation of nuclei. However, fibril elongation during agitation is limited by rapid consumption of the monomers^18^. As the amount of CB[6] increases in the INS solution (Fig. 3a), the lag phase increases in the assay. The fibril length increases with increased amounts of CB[6] in solution and a low polydispersity (PDI ~1.3–1.5) because the solution agitation was maintained (Supplementary Fig. S6). We suggest that 1) amounts of CB[6] above its solubility suppress monomer nucleation, 2) the high monomer:nucleus ratio is maintained by nucleation suppression, and 3) the preformed nuclei are elongated into long and homogeneous fibrils, while CB[6] hinders additional nucleation (Fig. 3b). During nucleation, the amount of CB[6] above its solubility limit determines the monomer:nucleus ratio that corresponds to the length of the formed INS fibrils (Fig. 3c).

CB[6] delays the nucleation of INS (Fig. 3a), but the fibrillation is not fully inhibited. We expect that INS in the insoluble state is dissociated from CB[6] and redissolved in the solution for more energetically favoured fibril formation^38^ when the active concentration of INS in the soluble state decreases upon fibril assembly (Fig. 1d). CB[6] induces a phase transfer of the CB[6]-guest complex into a kinetically trapped crystal^39^, which then slowly redissolves into the solution and recrystallizes as a thermodynamically stable state. Similarly, the reversible transfer of INS between an insoluble and a soluble state is expected; thereby the redissolved INS will serve as a monomer to interact with preformed nuclei for the elongation of INS fibrils. Our infrared spectra support this supposition, in that CB[6] does not participate in fibril formation (Supplementary Fig. S10).

We further examined whether CB[6] could modulate the fibrillation by interaction with the nuclei. Thus, an INS solution (50 μM) was incubated with agitation to prepare short fibrils that could behave as the nuclei; then, the solution was further incubated with CB[6] (Supplementary Fig. S11). CB[6] does not change the morphology of the preformed short fibrils. This result implies that CB[6] cannot induce the morphological change of self-assembled INS species. However, when INS monomers (50 μM) and CB[6] (500 μM) were simultaneously added to the preformed short fibrils, long INS fibrils were generated and the preformed short fibrils disappeared (Supplementary Fig. S11). This result supports that CB[6] does not disrupt the growth of the preformed short fibrils. Hence, we expect that self-assembled INS species, including small oligomers and nuclei, are involved in the fibril growth, rather than the direct interaction with CB[6].

### General applicability of CB[6] in controlling a range of amyloid fibrils

**Figure 4:**
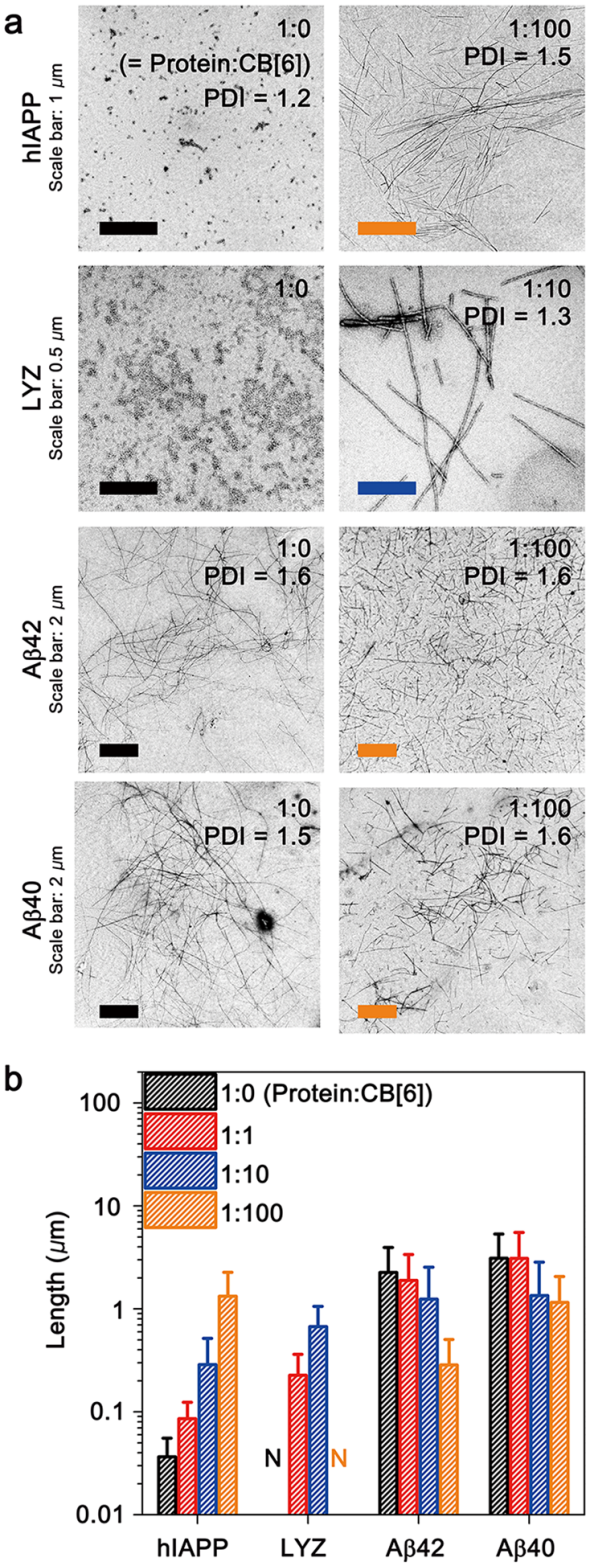
General applicability of CB[6] in the control of various amyloid fibrils. (**a**) TEM images of hIAPP, LYZ, Aβ42, and Aβ40 fibrils. (**b**) Lengths of amyloid fibrils controlled by CB[6]. The concentration of LYZ was 50 μM, and those of hIAPP, Aβ42, and Aβ40 were 10 μM. Numerical values of the fibril lengths and PDI are available in Supplementary Table S4.

The general applicability of CB[6] in controlling the fibril lengths was examined using several amyloid proteins (human islet amyloid polypeptide (hIAPP), hen egg white lysozyme (LYZ), amyloid-β 1–42 (Aβ42), and amyloid-β 1–40 (Aβ40)), whose core sequences are widely used in the design of functional amyloid fibrils (Fig. 4a)^40^. Since these amyloid proteins include Lys in their amino acid sequences, we expected that the interaction with CB[6] can modulate the fibrillation process of the proteins. The use of hIAPP without CB[6] under agitation generates short fibrils (35 ± 15 nm, PDI = 1.2). LYZ incubated without CB[6] under agitation forms small amorphous aggregates. Upon the addition of CB[6], the fibril lengths of hIAPP and LYZ increase as the protein:CB[6] ratio increases (PDI ~ 1.2–1.5, Fig. 4b). These results indicate that the fibril lengths of hIAPP and LYZ with low polydispersity can be controlled using agitation and CB[6]. Aβ42 and Aβ40 were also incubated at protein: CB[6] = 1:0 with agitation, whereby long fibrils (2.3 ± 1.7 μm and 3.1 ± 2.2 μm, respectively) with PDIs of 1.6 and 1.5, respectively, are formed (Fig. 4a). Increases in the amount of CB[6] in solution decrease the fibril lengths of Aβ, and the PDI values are maintained in the range of 1.5–2.1 (Fig. 4b). The trends for the fibril lengths of Aβ42 and Aβ40 are opposite of those for hIAPP and LYZ, and they show higher polydispersities.

Host-guest interactions between CB[6] and Lys of the proteins are confirmed by ESI-MS (Supplementary Fig. S12) and MS^2^ (Supplementary Fig. S13). In addition, the protein phase transfers by CB[6] are also confirmed (Supplementary Fig. S14). These results indicate that interactions with CB[6] are common for the four proteins; therefore, the differences in the fibrillation are due to their intrinsic properties. Thus, we compared the nucleation rates (*k*_nu_) of the proteins with the complexation rates of CB[6] with primary amines (*k*_CB[6]_). The values of *k*_CB[6]_ (~10^0^–10^2^ M^−1^ s^−1^)^41^ are higher than that of *k*_nu_ in INS fibrillation (~10^–2^ M^−1^ s^−1^), whereas *k*_CB[6]_ is on the same order of magnitude as *k*_nu_ for the Aβ peptides (~10^0^ M^−1^ s^−1^ for Aβ40, >10^0^ M^−1^ s^−1^ for Aβ42)^18, 42^. The fibrillation of hIAPP is approximately two orders of magnitude slower than that of Aβ40^43^, and LYZ fibrillation is slower than that for other proteins^44^. The ThT assay of Aβ40 fibrillation suggests that CB[6] is not able to interrupt the nucleation of Aβ40 (Supplementary Fig. S14), and in contrast to other proteins, the nucleation of Aβ peptides proceeds via their original pathway because the Aβ-CB[6] complexation is slower than the assembly of Aβ peptides. However, increasing the amount of CB[6] in the solution disrupts the protein-protein interactions of Aβ-CB[6] during fibril elongation. Thus, the lengths of the Aβ fibrils decrease in the presence of CB[6].

## Conclusion

We suggest that amyloid fibrils of various well-known amyloid proteins can be manipulated with desired lengths, ranging from tens of nanometres to nearly ten micrometres, and a low heterogeneity. This is the first demonstration of supramolecular manipulation that enables kinetic control of fibril assemblies by CB[6]-induced protein phase transfer. We believe that our approach based on the phase transfer of proteins will provide valuable insights into a rational strategy to obtain controlled functional amyloid fibrils.

### Data availability

All data generated or analysed during this study are included in this published article (and its Supplementary Information files).

## Electronic supplementary material

Supplementary Information
