# Supplementary material for: Nanoscale Control of Amyloid Self-Assembly Using Protein Phase Transfer by Host-Guest Chemistry
Source: Sci Rep. 2017 Jul 18;7:5710. doi: 10.1038/s41598-017-06181-4 (PMC5516018; doi:10.1038/s41598-017-06181-4)
Supplement: Supplementary file 1 — Supplementary Information [file 41598_2017_6181_MOESM1_ESM.pdf]

Supplementary Information for

Nanoscale Control of Amyloid Self-Assembly Using Protein Phase Transfer by Host-Guest Chemistry

Tae Su Choi,[a,b] *Hong Hee Lee,*[c] *Young Ho Ko,*[d] *Kwang Seob Jeong,*[a] *Kimoon Kim,*[b,d] *and Hugh I. Kim**[a,d]

Corresponding Author

*To whom correspondence should be addressed: hughkim@korea.ac.kr.

Experimental Section

Protein fibril preparation. Human recombinant insulin (INS, zinc-free), hen egg white lysozyme (LYZ), cucurbit[6]uril hydrate (CB[6]), and formic acid (FA) were purchased from Sigma-Aldrich (St. Louis, MO, USA). Human islet amyloid polypeptide and amyloid-β 1-40 peptide were purchased from Peptron (Daejeon, Republic of Korea). Amyloid-β 1-42 peptide was purchased from AnyGen (Jangheung, Republic of Korea). A protein stock solution was prepared at a concentration of 100 μM in 0.1% FA, and a 50 mM CB[6] stock solution was prepared in 50% FA. We prepared all solutions under acidic conditions because the net charges of amyloid proteins are positive in low pH, and CB[6] preferentially interacts with positively charged molecules.[1] In addition, INS is highly soluble under acidic conditions and is converted from multimeric states (dimer, tetramer, and hexamer) to the monomeric state.[2]

Stock solutions were transferred to 4-mL borosilicate glass vials and diluted to a final volume of 1 mL. Protein concentrations were adjusted to 50 μM for INS and LYZ and 10 μM for amyloid-β and human islet amyloid polypeptide to prevent aggregation prior to incubation in the 5% FA solution. All protein solutions were incubated at 50 °C for four days. Agitation was performed at 200 rpm. When we examined CB[6]-mediated fibrillation at a high temperature (60 °C), CB[6] was not effective for controlling the fibrillation process (Supplementary Fig. S15). In contrast, at a low temperature (40 °C), the fibrillation process itself was not promoted. The fibrillation process using CB[6] was best controlled at 50 °C.

CB[6] is hardly soluble in aqueous solutions; thus, above the CB[6] solubility limit, CB[6] settled to the bottom of the 4-mL vial. INS fibrils in the presence of CB[6] without agitation were not observed. We further tested the effect of changes in the %FA and temperature without agitation and confirmed that both factors were not significant for improving the homogeneity of the generated fibril lengths. A condition containing metal ions was excluded because the binding of metal ions to CB[6] could disrupt the control of the fibril lengths (Supplementary Fig. S16).

Sample treatment for TEM analysis. Formvar/carbon-coated copper grids (400-mesh) were purchased from Electron Microscopy Sciences (Hatfield, PA, USA). Aliquots of the incubated solution (5 μL) were spotted onto the grid for 3 min, and the spotted solution was then removed using a micropipette tip. Finally, a 1 wt% uranyl acetate solution in water (5 μL) was spotted onto the grid for 1 min. The grid was treated once for 5 s with 5% FA to remove any remaining CB[6]. The treated grids were analyzed using a JEM-1011 transmission electron microscope (TEM; JEOL, Japan) at the Biotech Center of Pohang University of Science and Technology (POSTECH) in Pohang, Republic of Korea and a Tecnai G2 F30ST (FEI, USA) at the Korea Basic Science Institute (KBSI) in Seoul, Republic of Korea.

Fibril analysis. Measurements of the fibril lengths and thicknesses were performed using the KLONK image measurement software (Denmark). The fibril lengths and thicknesses were defined as the end-to-end distance and the width of the fibril, respectively. The lengths of 500–2000 fibril species and the thicknesses of 200 fibril species were measured. The average lengths of the fibrils are plotted on a logarithmic scale. The PDI values were

calculated using the mathematical definition of PDI described in the Supplementary Text (*vide infra*).

Thioflavin T (ThT) assays. ThT assays were performed to obtain information about the fibrillation kinetics. A ThT stock solution (1 mg/mL) was prepared. An aliquot of incubated protein solution (25 μL), ThT stock solution (25 μL), and 5% FA (450 μL) were mixed prior to the fluorescence measurements. The excitation wavelength of ThT was set to 452 nm and the emission was scanned from 460 to 490 nm. The emission at 482 nm was used for monitoring the fibrillation kinetics. Since the fluorescence intensity of ThT increases upon complexation with CB[6], the baseline was corrected using a ThT-CB[6] solution without proteins. All data points were normalized using the height of the stationary phase.

Isothermal titration calorimetry (ITC). ITC experiments were performed using a VP-ITC calorimeter (MicroCal, Worcestershire, UK) to obtain an equilibrium association constant (K_a) for the interaction between INS and CB[6]. INS and CB[6] solutions were freshly prepared and degassed prior to each measurement. The reference cell was filled with 50% FA, and an INS solution in 50% FA (100 μM) was loaded into the ITC cell. A CB[6] solution (1 mM) was injected 30 times through the ITC syringe. The interval of each injection was 3 min. The ITC cell was stirred at 502 rpm and maintained at 25 °C. The heats of dilution by CB[6] were subtracted for analysis. All results were treated using the 'single-set-of-sites' model internally stored in Origin 7.0 (associated with the VP-ITC calorimeter).

Ultraviolet-visible spectroscopy. Protein solutions with and without CB[6] were freshly prepared to measure the protein concentration in the solutions. The solutions were gently

mixed for 1 min and centrifuged for 15 min at 18,000×g at 4 °C. The UV-visible spectra of the supernatants of the centrifuged solutions were obtained using a HP5520 UV-Vis spectrophotometer (HP, Santa Clara, CA, USA). The concentration of INS was calculated using the extinction coefficient of 5,960 $M^{-1}\cdot cm^{-1}$ at 280 nm. The concentrations of human islet amyloid polypeptide and amyloid-β were calculated using the extinction coefficient of 1,490 $M^{-1}\cdot cm^{-1}$ at 280 nm. For LYZ, the extinction coefficient of 37,970 $M^{-1}\cdot cm^{-1}$ at 280 nm was used.

Electrospray ionization mass spectrometry. Electrospray ionization mass spectrometry (ESI-MS) and tandem mass spectrometry (MS^2) using collision-induced dissociation (CID) were performed using an LTQ Velos dual ion trap mass spectrometer (Thermo Scientific, Waltham, MA, USA) equipped with an ESI source in positive ion mode (electrospray voltage: 3.5 kV, capillary temperature: 150 °C). CID analysis of $[LYZ\text{-}CB[6]]^{9+}$ was further performed using a Synapt G2 HDMS quadrupole time of flight (qTOF) instrument with travelling ion mobility mass spectrometry (TWIMS; Waters, Milford, MA, USA; electrospray voltage: 1.5 kV, capillary temperature: 80 °C).

Infrared spectroscopy. Infrared (IR) spectroscopy was performed using a Nicolet iS10 spectrometer (Thermo Scientific, Waltham, MA, USA). A total of 128 spectra for each sample were obtained with a resolution of 4 cm^{-1}, which were averaged to generate a single spectrum. Fibril samples were prepared following the procedure described in the 'fibril sample preparation' section above. Unwashed samples were directly cast onto a silicon wafer and fully dried for a day under air. Washed samples were loaded into an Amicon 0.5-mL 100k centrifugal filter (Millipore, Billerica, MA, USA) and then washed three times with 5% FA to remove the excess CB[6] and non-fibrillar species in the samples

before being loaded onto silicon wafers. All IR spectra were normalized using the highest peak intensity in the range of 1500–1900 cm^{-1}.

Solution Small-Angle X-ray Scattering (SAXS). All SAXS measurements were performed at the 4C SAXS II beamline of the Pohang Accelerator Laboratory (PAL) in Pohang University of Science and Technology (POSTECH). The concentration of INS was measured as 0.76 mg/mL in 1% FA. The sample-to-detector distance was set to 2 m, and the temperature was fixed at 20 °C. Each measurement was performed four times using fresh INS samples. The scattering patterns were recorded six times (5 s each with 1-s intervals). SAXS measurements of standard proteins (trypsin, human serum albumin, and concalvalin A) were performed following the same procedures. After the measurements, the concentrations of the standard proteins were also measured. The radius of gyration (R_g) and molecular weight (M_w) calibration using the zero-angle scattering intensity ($I(0)$) were estimated using a procedure in the literature.[3]

Supplementary text

Polydispersity index. The polydispersity index (PDI)[4] is the ratio of the mass average degree of polymerization ($\overline{L_w}$) to the number average degree of polymerization ($\overline{L_n}$). If the polymer is ideally monodisperse, PDI = 1. The PDI value is usually larger than 1 because of the heterogeneous distribution of the polymers. In the present study, the length of the amyloid fibril was adopted as the degree of polymerization because its length was linearly proportional to the number of protein monomers within the fibril.

$$\overline{L_w} = \frac{l_1^2 n_1 + l_2^2 n_2 + \cdots + l_m^2 n_m}{l_1 n_1 + l_2 n_2 + \cdots + l_m n_m}$$

$$\overline{L_n} = \frac{l_1 n_1 + l_2 n_2 + \cdots + l_m n_m}{n_1 + n_2 + \cdots + n_m}$$

$$\mathrm{PDI} = \overline{L_w}/\overline{L_n}$$

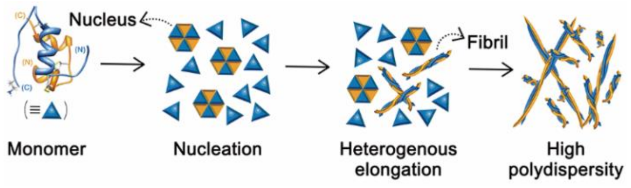

Supplementary Figure S1. Schematic representation of the nucleation-growth mechanism. Monomeric proteins self-assemble to the nucleus during the initial stage. The nucleus is elongated to form an amyloid fibril. The heterogeneity of the amyloid assembly is propagated during nucleation because nuclei formation is not simultaneous.

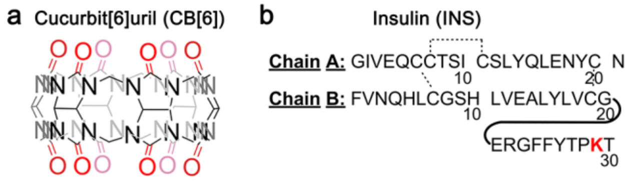

Supplementary Figure S2. (a) Structure of CB[6]. (b) Structure of INS, which contains a single Lys residue (marked in red).

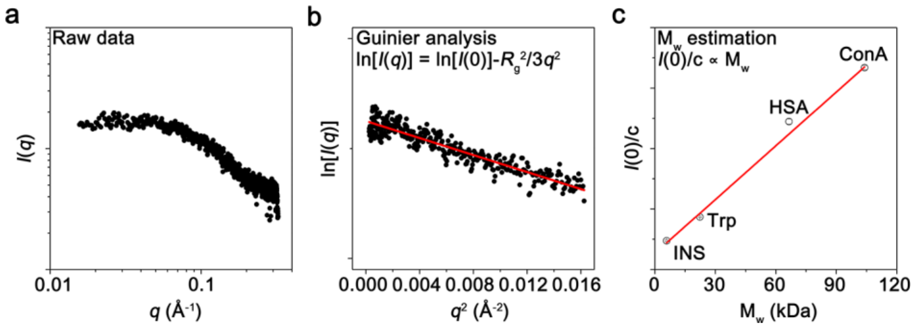

Supplementary Figure S3. (a) Raw data of the INS scattering pattern in 1% FA. The concentration of INS was 0.76 mg/mL (~131 μM). (b) Estimation of the R_g value of INS in 1% FA using the Guinier approximation. (c) Estimation of the molecular weight of INS in 1% FA using commercially available standard proteins (Trp = trypsin, HSA = human serum albumin, and ConA = concavalin A). When the M_w value of INS was set to the value in the monomeric state, the extrapolation from the standard proteins to INS was well matched to the linear fit.

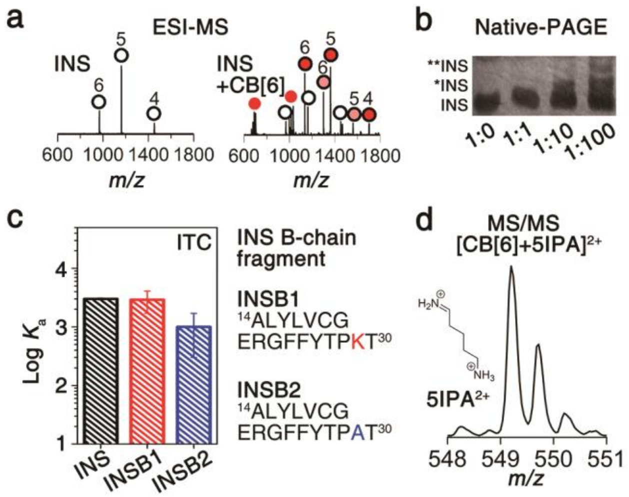

Supplementary Figure S4. (a) ESI-MS spectra of INS and INS-CB[6]. INS (white circle) shows the 4+ to 6+ charge states. INS-CB[6] complex peaks were also detected with the same charge states. Numbers on the circles indicate the charge state of INS and INS-CB[6]. A 1:1 complex of INS-CB[6] (the circle filled with red) was the major species observed, and further complexation with CB[6] (INS-2CB[6]) was also detected as a minor portion (the circle filled with pink). Minor interactions between INS and CB[6] appear in the presence of positively charged residues on the protein (N5-terminus, histidine, and arginine). (b) Native-PAGE of the INS solution. Asterisks indicate the number of CB[6] units bound to INS. The concentration of INS was 50 μM. (c) K_a values of CB[6] with INS and its B-chain fragments. INS and its B-chain fragments (INSB1) show similar K_a values of 3.0×10^3 and 2.9×10^3 M^{-1}, respectively. To examine the effects of Lys in INSB1, Lys was mutated to alanine in INSB2, and the K_a of INSB2 decreased to 1.0×10^3 M^{-1}. Thus, the ITC results indicate that Lys is crucial for the interaction between INS and CB[6]. We made the assumption in these measurements that the peptide fragment of INS could reflect the environment of Lys in the full protein because Lys is located in the random coil region (C-terminus of INS B-chain).[5,6] (d) Lys-selected fragmentation with CB[6] at *m/z* 549.[7]

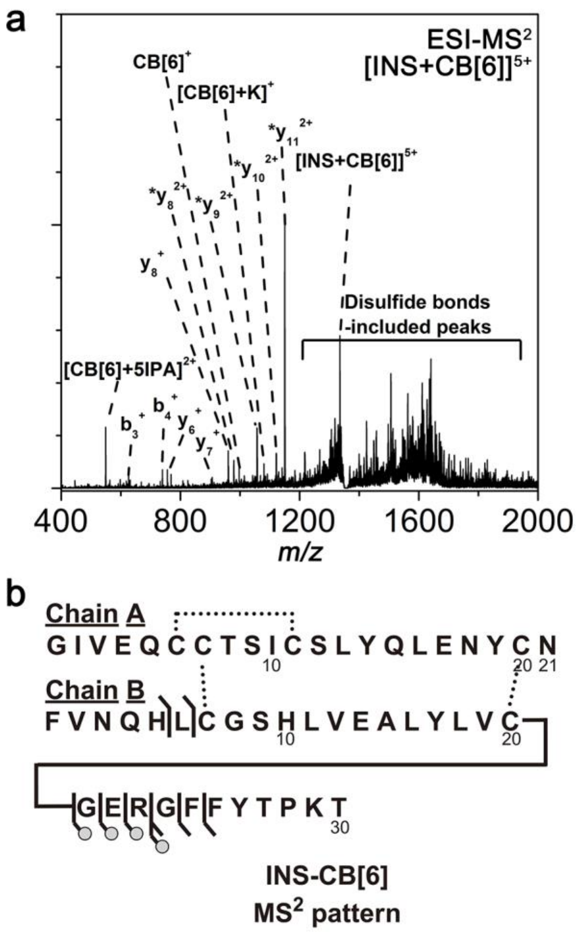

Supplementary Figure S5. (a) ESI-MS^2 spectrum of the INS-CB[6] complex. The asterisk indicates the fragment complexed with CB[6]. (b) MS^2 fragmentation pathway of the INS-CB[6] complex. The bar with filled circles indicates the INS fragment complexed with CB[6], and the bar without circles shows the INS fragment only. The fragmentation pattern of the complex shows y-type fragments of the INS B-chain C-terminus, and b-type fragments of the INS B-chain N-terminus; y-type fragments of the C-terminus mostly complexed with CB[6], but b-type fragments of the N-terminus did not form a complex with CB[6].

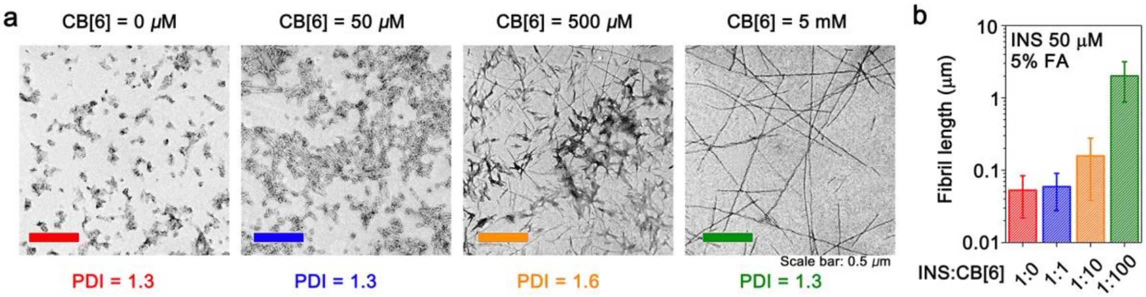

Supplementary Figure S6. (a) TEM images of INS fibrils formed in 5% FA with various amounts of CB[6] in solution. (b) Average lengths of INS fibrils. The concentration of INS was 50 μM, and the agitation was 200 rpm.

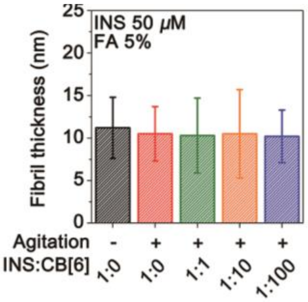

Supplementary Figure S7. Average thicknesses of the INS fibrils measured from TEM images. No significant changes within the error ranges were observed.

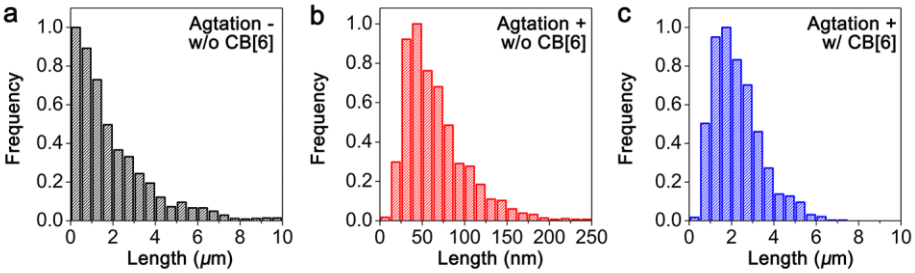

Supplementary Figure S8. Length distribution of INS fibrils formed (a) without agitation and CB[6], (b) with agitation and without CB[6], and (c) with agitation and CB[6]. The concentration of INS was 50 μM, and the agitation was 200 rpm. The concentration of CB[6] was 5 mM. All INS solutions were incubated in 5% FA.

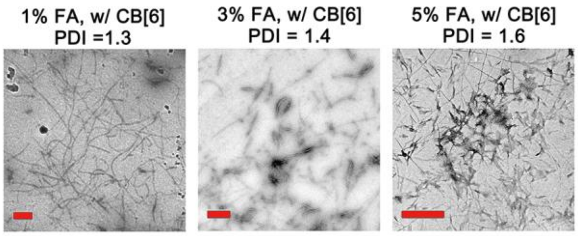

Supplementary Figure S9. INS fibrils formed in various %FA. The concentrations of INS and CB[6] were 50 μM and 500 μM. All solutions were agitated at 200 rpm.

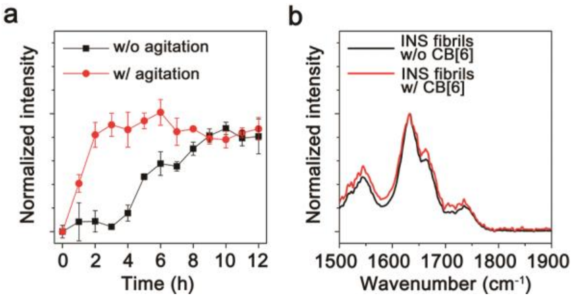

Supplementary Figure S10. (a) ThT assay for the kinetics of INS fibrillation with and without agitation in solution. The concentration of INS was 50 μM. (b) IR spectra of INS fibrils after washing away CB[6] and non-fibrillar species.

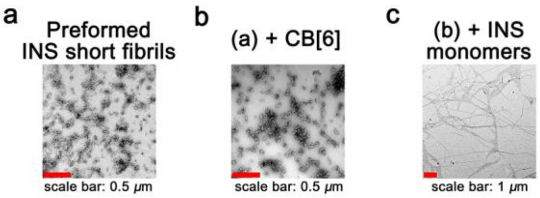

Supplementary Figure S11. (a) Morphology of preformed INS short fibrils after the incubation of 50 μM INS with 200 rpm agitation at 50 °C in 0.1% FA. (b) Morphology of the preformed short fibrils after incubation with CB[6] (5 mM). (c) Morphology of INS fibrils after the incubation with the preformed short fibrils (1 μM), CB[6] (500 μM), and INS monomers (50 μM) in 0.1% FA.

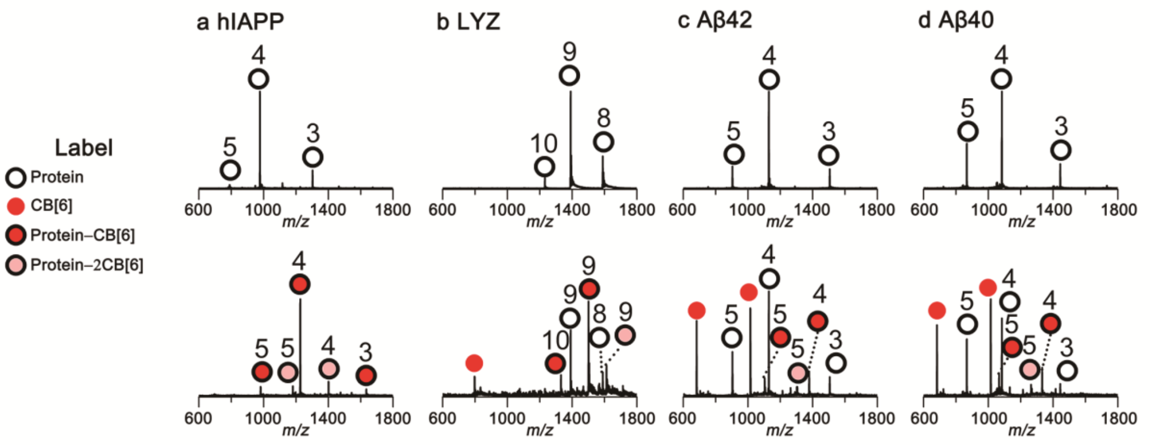

Supplementary Figure S12. ESI-MS spectra of proteins in the absence and presence of CB[6]. Numbers indicate the charge state of proteins. (a) ESI-MS spectra of hIAPP and hIAPP-CB[6]. hIAPP shows 3+ to 5+ charge states, and hIAPP-CB[6] complexes were also detected with the same charge states. The 1:1 complex of hIAPP-CB[6] was the major species, and further complexation with CB[6] (hIAPP-2CB[6]) was also detected as a minor fraction. (b) ESI-MS spectra of LYZ and LYZ-CB[6]. LYZ shows 8+ to 10+ charge states, and LYZ-CB[6] complexes were also detected with the same charge states. The 1:1 complex of the LYZ-CB[6] complex was the major species, and further complexation with CB[6] (LYZ-2CB[6]) was also detected as a minor fraction. ESI-MS spectra of (c) Aβ42 and (d) Aβ40-CB[6] complexes. Aβ peptides show 3+ to 5+ charge states, and Aβ-CB[6] complexes were also detected with the same charge states. The 1:1 complex of the Aβ-CB[6] complex was the major species, and further complexation with CB[6] (Aβ-2CB[6]) was also detected as a minor fraction.

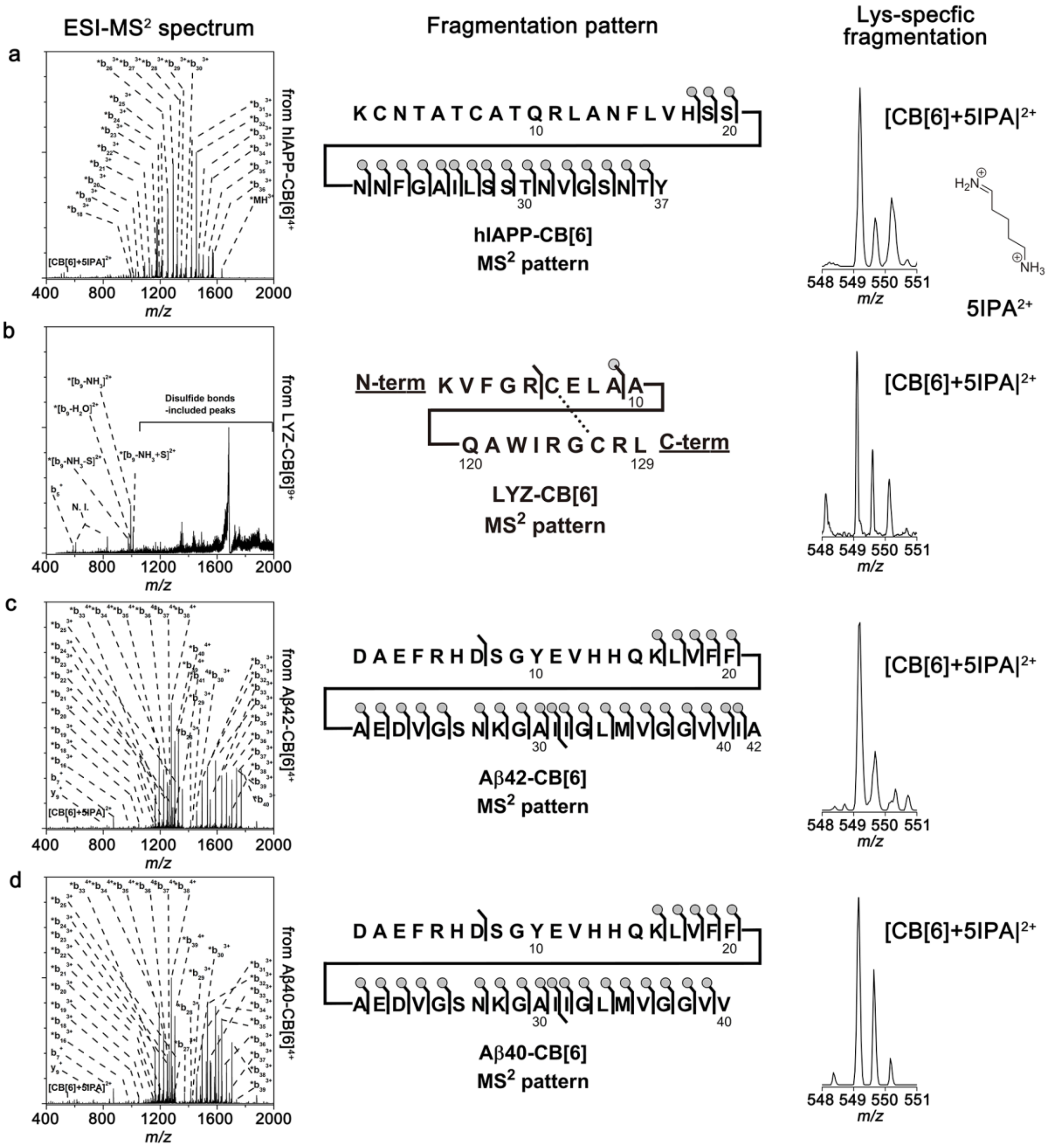

Supplementary Figure S13. (a) ESI-MS^2 spectrum of the hIAPP-CB[6] complex. The asterisk indicates the fragment complexed with CB[6]. The bar with filled circle indicates the hIAPP fragment complexed with CB[6], and the bar without the circle indicates the hIAPP fragment only. Lys-selected fragmentation with CB[6] at *m/z* 549. (b) ESI-MS^2 spectrum of the LYZ-CB[6] complex. The Lys-selected fragment of LYZ was obtained using a qTOF instrument (Synapt G2) because the molecular weight of LYZ was too large to exert collision energy in the ion trap (LTQ Velos). ESI-MS^2 spectra of (c) Aβ42-CB[6] and (d) Aβ40-CB[6] complexes. These results indicate that Lys residues are the most probable binding sites for CB[6] on the proteins.

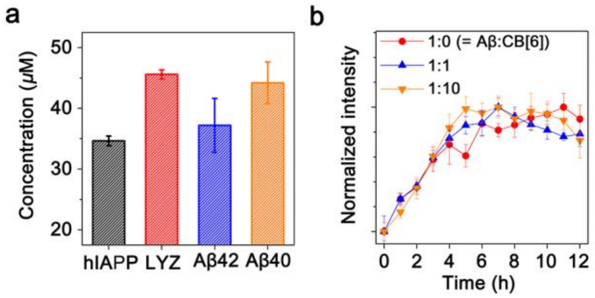

Supplementary Figure S14. (a) Concentrations of proteins in the 5% FA supernatant solution. The protein and CB[6] concentrations were 50 μM and 5 mM. (b) ThT assay of Aβ40 fibrillation. The Aβ40:CB[6] = 1:100 ratio was excluded because of light scattering by CB[6]. The Aβ40 solution was incubated at 50 °C with agitation at 200 rpm. The concentration of Aβ40 for the ThT assay was 10 μM.

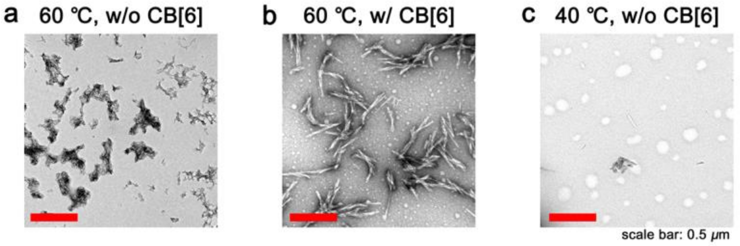

Supplementary Figure S15. (a) TEM image of an incubated INS solution (50 μM) with 200 rpm agitation at 60 °C in 5% FA. Short fibrils were abundant in this condition. (b) TEM image of an incubated INS solution (50 μM) with 5 mM CB[6] and 200 rpm agitation at 60 °C in 5% FA. Fibril lengths increased by CB[6], but the effect of CB[6] was less dramatic in this condition, compared to 50 °C. (c) TEM image of an incubated INS solution (50 μM) with 200 rpm agitation at 40 °C in 5% FA. INS fibrils were not abundantly formed at 40 °C.

Temperature is crucial for the kinetics of amyloid fibrillation, in that the activation energy for protein folding/unfolding is related to the melting temperature of the proteins (the melting temperature of INS is approximately 60 °C).[8] When we examined CB[6]-mediated fibrillation at a high temperature (60 °C), CB[6] was not effective for controlling the fibrillation process. In contrast, at a low temperature (40 °C), the fibrillation process itself was inhibited. This seems to be because CB[6] cannot kinetically modulate the self-assembly if the nucleation rate is too fast; if the nucleation rate is too slow, the fibrillation process itself is inhibited.

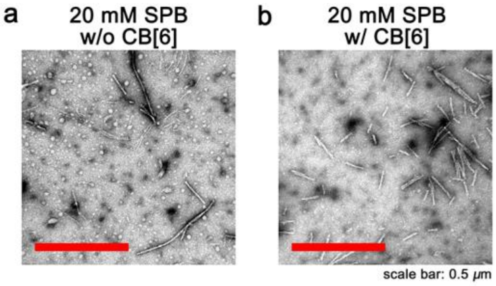

Supplementary Figure S16. (a) TEM image of an incubated INS solution (10 μM) with 200 rpm agitation at 50 °C in a 20 mM sodium phosphate buffer (SPB). (b) TEM image of an incubated INS solution (10 μM) with 1 mM CB[6] and 200 rpm agitation at 50 °C in 20 mM SPB. The concentration of INS was adjusted from 50 μM to 10 μM because the solubility of INS is limited to 10 μM in 20 mM SPB at pH 7.4.

The interaction between sodium and CB[6][9] can compete with that between CB[6] and Lys of INS. Thus, we expect that the Lys-CB[6] interaction is disrupted in the presence of sodium ions; thereby, CB[6] cannot control the fibrillation process of INS.

Supplementary Table S1. Solubility of CB[6] upon variations in %FA (*v*/*v*).[10]

%FA	mM
0	0.02
5	0.08
10	0.38
15	0.55
20	1.18
30	6.47

Supplementary Table S2. Average fibril lengths and PDIs of INS fibrils in Figs. 3b and Supplementary Fig. S4. The concentration of INS was 50 µM.

Agitation<br>INS:CB[6]	-<br>1:0	+<br>1:0	+<br>1:1	+<br>1:10	+<br>1:100
Avg length	2.3±3.0 µm	53±31 nm	59±31 nm	0.16±0.12 µm	2.0±1.1 µm
PDI	2.8	1.3	1.3	1.6	1.3

Supplementary Table S3. Average fibril lengths and PDIs of INS fibrils in Fig. 3d. The concentration of INS was 50 µM, and the ratio of INS:CB[6] was set to 1:10. Agitations of 200 rpm were INS solutions.

%FA		0.1%	1%	3%	5%	15%
w/ CB[6]	Avg length	9.8±5.1 µm	2.1±1.2 µm	1.3±0.9 µm	0.16±0.12 µm	0.10±0.07 µm
	PDI	1.3	1.3	1.4	1.6	1.5
w/o CB[6]	Avg length	43±36 nm	46±20 nm	52±23 nm	53±31 nm	37±19 nm
	PDI	1.5	1.2	1.2	1.3	1.3

Supplementary Table 4. Average fibril lengths and PDI of protein fibrils in Figure 5 Concentrations of hIAPP and Aβ were 10 µM, and that of LYZ was 50 µM.

hIAPP:CB[6]	1:0	1:1	1:10	1:100
Avg length<br>PDI	35±15 nm<br>1.2	86±38 nm<br>1.2	0.29±0.23 µm<br>1.3	1.3±0.9 µm<br>1.5
LYZ:CB[6]	1:0	1:1	1:10	1:100
Avg length<br>PDI	Worm-like aggregates	0.23±0.14 µm<br>1.4	0.67±0.39 µm<br>1.3	No fibril
Aβ42:CB[6]	1:0	1:1	1:10	1:100
Avg length<br>PDI	2.3±1.7 µm<br>1.6	1.9±1.5 µm<br>1.6	1.2±1.3 µm<br>2.1	0.28±0.22 µm<br>1.6
Aβ40:CB[6]	1:0	1:1	1:10	1:100
Avg length<br>PDI	3.1±2.2 µm<br>1.5	3.1±2.4 µm<br>1.6	1.6±1.5 µm<br>1.9	1.2±0.9 µm<br>1.6

References

1. Barrow, S.J., Kasera, S., Rowland, M.J., del Barrio, J. & Scherman, O.A. Cucurbituril-Based Molecular Recognition. *Chem. Rev.* **115**, 12320-12406 (2015).
2. Hua, Q.X. & Weiss, M.A. Mechanism of insulin fibrillation - The structure of insulin under amyloidogenic conditions resembles a protein-folding intermediate. *J. Biol. Chem.* **279**, 21449-21460 (2004).
3. Putnam, C.D., Hammel, M., Hura, G.L. & Tainer, J.A. X-ray solution scattering (SAXS) combined with crystallography and computation: defining accurate macromolecular structures, conformations and assemblies in solution. *Q. Rev. Biophys.* **40**, 191-285 (2007).
4. Gilbert, R.G. et al. Dispersity in polymer science (IUPAC Recommendations 2009) (vol 81, pg 351, 2009). *Pure Appl. Chem.* **81**, 779-779 (2009).
5. Lee, H.H. et al. Supramolecular Inhibition of Amyloid Fibrillation by Cucurbit[7]uril. *Angew. Chem. Int. Ed.* **53**, 7461-7465 (2014).
6. Chinai, J.M. et al. Molecular Recognition of Insulin by a Synthetic Receptor. *J. Am. Chem. Soc.* **133**, 8810-8813 (2011).
7. Heo, S.W. et al. Host-Guest Chemistry in the Gas Phase: Selected Fragmentations of CB[6]-Peptide Complexes at Lysine Residues and Its Utility to Probe the Structures of Small Proteins. *Anal. Chem.* **83**, 7916-7923 (2011).
8. Dzwolak, W., Ravindra, R. & Winter, R. Hydration and structure-the two sides of the insulin aggregation process. *Phys. Chem. Chem. Phys.* **6**, 1938-1943 (2004).
9. Noh, D.H., Lee, S.J.C., Lee, J.W. & Kim, H.I. Host-Guest Chemistry in the Gas Phase: Complex Formation of Cucurbit[6]uril with Proton-bound Water Dimer. *J. Am. Soc. Mass Spectrom.* **25**, 410-421 (2014).
10. Buschmann, H.J., Jansen, K., Meschke, C. & Schollmeyer, E. Thermodynamic data for complex formation between cucurbituril and alkali and alkaline earth cations in aqueous formic acid solution. *J. Solution Chem.* **27**, 135-140 (1998).
